# Impacts of different climate change regimes and extreme climatic events on an alpine meadow community

**DOI:** 10.1038/srep21720

**Published:** 2016-02-18

**Authors:** Juha M. Alatalo, Annika K. Jägerbrand, Ulf Molau

**Affiliations:** 1 Department of Biological and Environmental Sciences, College of Arts and Sciences, Qatar University, P.O. Box 2713, Doha, Qatar; 2 VTI, Swedish National Road and Transport Research Institute, Box 55685, 102 15 Stockholm, Sweden; 3 Department of Biological and Environmental Sciences, University of Gothenburg, PO Box 461, SE-405 30 Gothenburg, Sweden

## Abstract

Climate variability is expected to increase in future but there exist very few experimental studies that apply different warming regimes on plant communities over several years. We studied an alpine meadow community under three warming regimes over three years. Treatments consisted of (a) a constant level of warming with open-top chambers (ca. 1.9 °C above ambient), (b) yearly stepwise increases in warming (increases of ca. 1.0, 1.9 and 3.5 °C), and (c) pulse warming, a single first-year pulse event of warming (increase of ca. 3.5 °C). Pulse warming and stepwise warming was hypothesised to cause distinct first-year and third-year effects, respectively. We found support for both hypotheses; however, the responses varied among measurement levels (whole community, canopy, bottom layer, and plant functional groups), treatments, and time. Our study revealed complex responses of the alpine plant community to the different experimentally imposed climate warming regimes. Plant cover, height and biomass frequently responded distinctly to the constant level of warming, the stepwise increase in warming and the extreme pulse-warming event. Notably, we found that stepwise warming had an accumulating effect on biomass, the responses to the different warming regimes varied among functional groups, and the short-term perturbations had negative effect on species richness and diversity

A growing number of studies have shown that a poleward movement of plant and animals is occurring. Although this trend has often been attributed to global warming, a simple northward movement of species cannot always be linked to climate change^1^. Climate change may also affect interspecific interactions, including mutualism between animals and plants^2^. However, the majority of existing studies are not evenly distributed among taxa or geography, and Europe and North America are commonly the source of these studies^3^. In the future, extreme climatic events, such as droughts, floods, heavy rainfall and heat waves, will become more common and more severe^4^, which may impact species as well as whole ecosystems^5^. How vegetation responds to extreme climatic events may depend on many factors^5^, such as functional diversity^6^,^7^, species diversity^8^, timing during succession, and various environmental factors^9^. Climate change is already causing an increasing number of ecosystems to encounter novel climatic events. In some cases, plant communities and ecosystems may switch to alternative regimes in response to a single climate event^10^,^11^. Heat waves have been observed to cause peat moss die-offs in the genus *Sphagnum*^12^. The timing of the climatic event is also of consequence; however, the consequences may differ among species in the same plant community, for example, a study found a negative impact on the net photosynthetic rate of the bryophyte *Hylocomium splendens,* whereas the lichen *Peltigera aphthosa* was unaffected by experimentally imposed winter warming^13^.

Organisms in polar and alpine ecosystems are thought to be at high risk to be affected by climate change as the temperatures remain above freezing for a very short summer season. Thus, a vast number of experimental studies using open-top chambers (OTCs) have been performed in these ecosystems to simulate climate change. These studies cover a wide range of taxa, from singular species to the community-level responses of vascular plants, bryophytes, lichens, arthropods, bacteria, and fungi^14^,^15^,^16^,^17^,^18^,^19^,^20^. In most studies, the focus of experimental climate change has centred on vascular plants^21^,^22^,^23^; however, bryophytes and lichens play an important role in arctic and subarctic vegetation communities, and their relative influence on cover, biomass, and nutrient cycling tend to increase with latitude^24^. Furthermore, these taxa have been shown to affect important processes, such as the recruitment of vascular plants^25^ and permafrost stability^26^,^27^,^28^.

Most studies using OTCs have only applied constant warming. However, constant warming might not be the most realistic simulation of future climate change, which is thought to be better represented by a more variable climate with more frequent and extreme climatic events. As there have been few experimental warming studies attempting to distinguish among the impacts of different regimes for climate warming and climatic events in alpine and arctic regions, there is a knowledge gap regarding how different climate change projections may affect plant communities in severe environments. A study on bryophyte and lichen communities in alpine Sweden incorporated three different warming regimes (constant warming for three years, a stepwise increase in warming over years, and a single season of pulse warming). The impact on community structure, functional groups, and species-specific responses of bryophytes and lichens revealed that acrocarpous bryophytes responded in a positive way to a season of extreme warming, whereas pleurocarpous bryophytes (except one species, *Tomentypnum nitens*), *Sphagnum* spp., and lichens were largely resilient to the different experimental warming regimes^29^. A laboratory study that exposed the bryophyte *Pleurozium schreberi*, which originated from eight different altitudinal sites, to three different temperature treatments found that the responses can vary among sites within a species, which indicates the difficulty in generalising the results from single-location studies^30^. An experiment imposing an extreme heat event in the High-Arctic Greenlandic tundra showed that vascular plants responded positively at first but deteriorated after the exposure^31^. In a second study at a Low-Arctic site in Greenland, researchers found more species-specific responses to two consecutive heat waves^32^. In another study at Disko Island, Greenland, subjecting the site to a heat wave over 13 days by infrared irradiation and incorporating a soil drought, researchers found contrasting responses: one species (*Polygonum vivipara*) was never stressed, a second species (*Salix arctica*) was stressed during the warming, and two species (*Pyrola grandiflora* and *Carex bigelowii*) exhibited a delayed response, which supports the hypothesis that responses may vary among species^33^.

In the present study, we aimed to distinguish among the impacts of constant warming (i.e., normal OTC perturbation), stepwise warming (warming that is successively raised stepwise over years), and pulse warming (one summer event of high warming to simulate a climatic event) on the abundance, biomass and community hierarchy of vascular plants and on total diversity (vascular plants, bryophytes and lichens). We have previously reported the impact on the community structure, functional groups and species-specific responses of bryophytes and lichens^29^. The following questions were addressed: (1) Are the responses to the standard OTC warming similar to the responses to stepwise and pulse warming? (2) Are the responses to stepwise warming and pulse warming different from each other? Specifically, we hypothesised that pulse warming would have the largest first-year effect compared to the other perturbations and that the stepwise increase in warming over the years would have the largest third-year effect.

## Results

### Impacts on canopy layer

**Figure 1:**
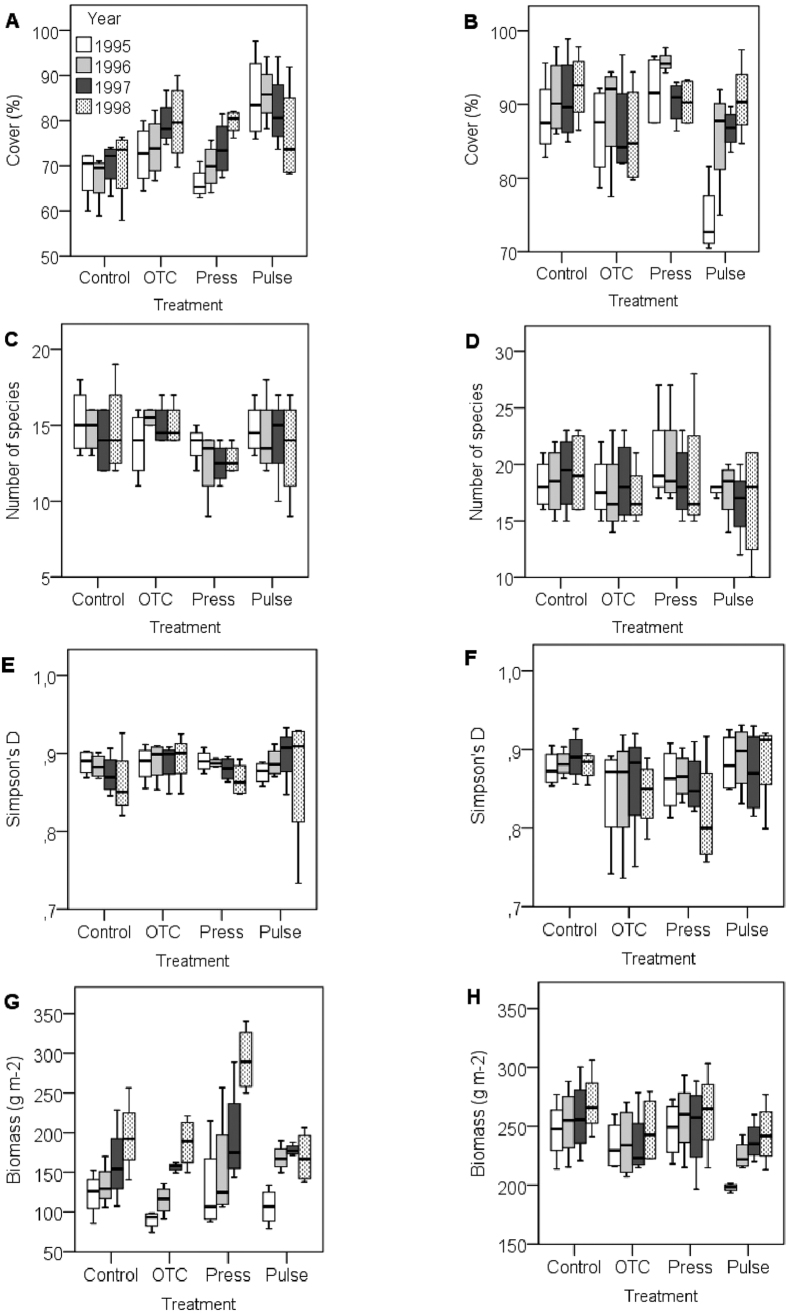
Boxplots of responses for the canopy layer and bottom layer in the alpine meadow. (**A**) Cover (%) in the canopy layer, (**B**) cover (%) in the bottom layer, (**C**) number of species in the canopy layer, (**D**) number of species in the bottom layer, (**E**) Simpsons diversity index (Simpson’s D) in the canopy layer, (**F**) Simpsons D in the bottom layer, (**G**) biomass (g/m^2^) in the canopy layer, and (**H**) biomass (g/m^2^) in the bottom layer. Treatments: control (Control), constant warming enhancement using open-top chambers (OTC), a stepwise increase in the magnitude of warming (Press) and a single-summer high-impact warming event (Pulse). Boxplots show the 10^th^ to 90^th^ percentiles of the data; n = 4 plots per treatment.

**Figure 2:**
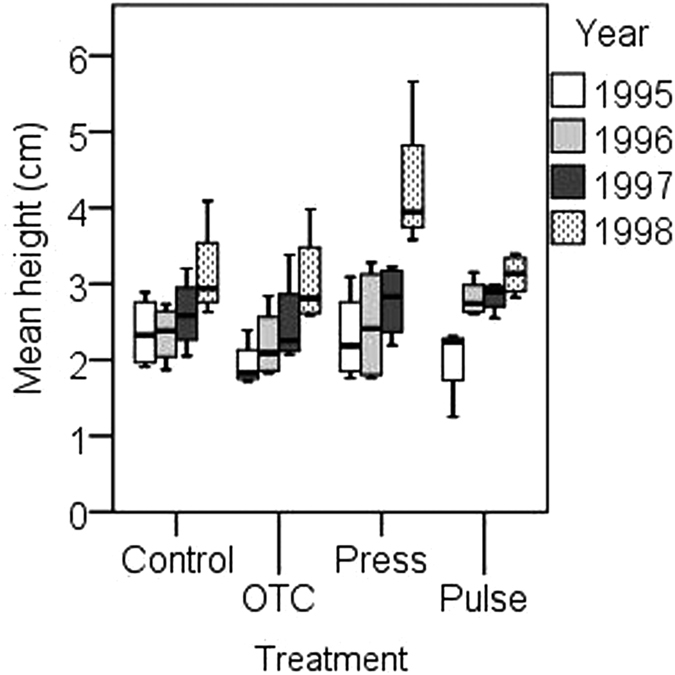
Boxplots of responses for mean height (cm) of the canopy layer in the alpine meadow. Treatments: control (Control), constant warming enhancement using open-top chambers (OTC), a stepwise increase in the magnitude of warming (Press) and a single-summer high-impact warming event (Pulse). Boxplots show the 10^th^ to 90^th^ percentiles of the data; n = 4 plots per treatment.

The experimental perturbations had a significant effect on cover, number of species, biomass, and plant height of the canopy layer but not on Simpson’s D (Table 1, Figs 1 and 2, Supplementary Dataset S1). Where the different warming treatments caused contrasting responses, the OTCs and stepwise warming had a positive effect on cover that increased over the years, whereas the pulse treatment seemed to cause the cover to decrease over the years (Fig. 1). However, plant height and biomass increased in the stepwise warming (press) treatment over the years, and species numbers tended to decline (Figs 1 and 2). A significant influence of years was found in biomass and height in the canopy layer. There were also various significant interactions between treatments and years with respect to cover, biomass, and height of the canopy layer (Table 1, Figs 1 and 2).

### Impacts on the bottom layer

In the bottom layer, the treatments had a significant effect on cover, number of species, Simpson’s D, and biomass (Table 1, Fig. 1). The different treatments caused different responses in cover: pulse treatments caused an increase over the years, whereas the stepwise warming caused an initial increase before returning to the starting level, and the OTCs caused no response (Fig. 1). Biomass tended to increase in response to all treatments over the years (Fig. 1). The pulse treatment tended to have a negative impact on the number of species (Fig. 1), and the stepwise warming tended to have a negative impact on Simpson’s D (Fig. 1). A significant positive influence of years was observed for cover and biomass in the bottom layer (Table 1, Fig. 1). Specific significant interactions between treatment and years (1995) were found for cover in the bottom layer (Table 1, Fig. 1).

### Impact on plant functional groups

**Figure 3:**
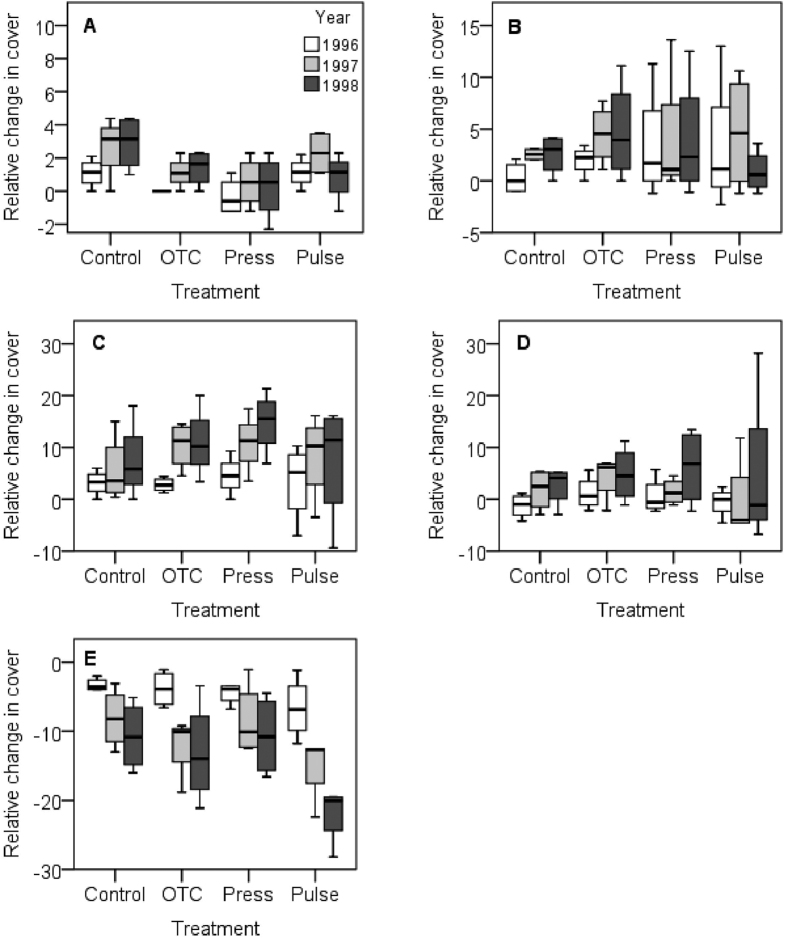
Boxplots of responses in relative change in cover in plant functional groups in the alpine meadow. (**A**) Cushions, (**B**) deciduous shrubs, (**C**) evergreens, (**D**) forbs, and (**E**) graminoids. Treatments: control (Control), constant warming enhancement using open-top chambers (OTC), a stepwise increase in the magnitude of warming (Press) and a single-summer high-impact warming event (Pulse). Boxplots show the 10^th^ to 90^th^ percentiles of the data; n = 4 plots per treatment.

**Figure 4:**
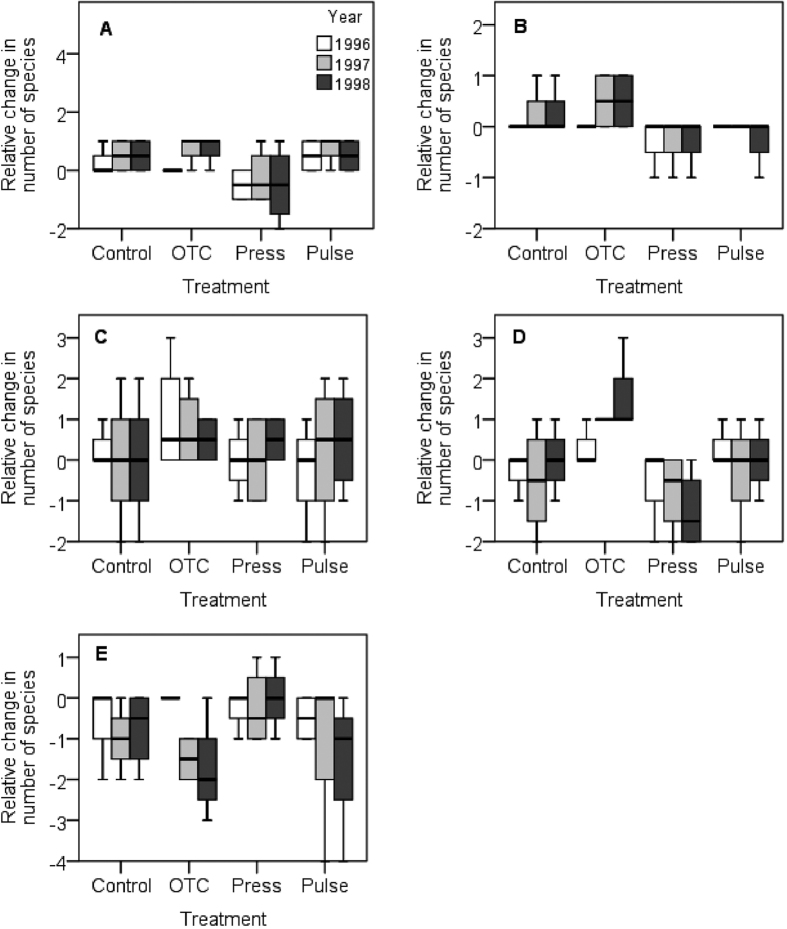
Boxplots of responses in relative change in the number of species in plant functional groups in the alpine meadow. (**A**) Cushions, (**B**) deciduous shrubs, (**C**) evergreens, (**D**) forbs, and (**E**) graminoids. Treatments: control (Control), constant warming enhancement using open-top chambers (OTC), a stepwise increase in the magnitude of warming (Press) and a single-summer high-impact warming event (Pulse). Boxplots show the 10^th^ to 90^th^ percentiles of the data; n = 4 plots per treatment.

**Figure 5:**
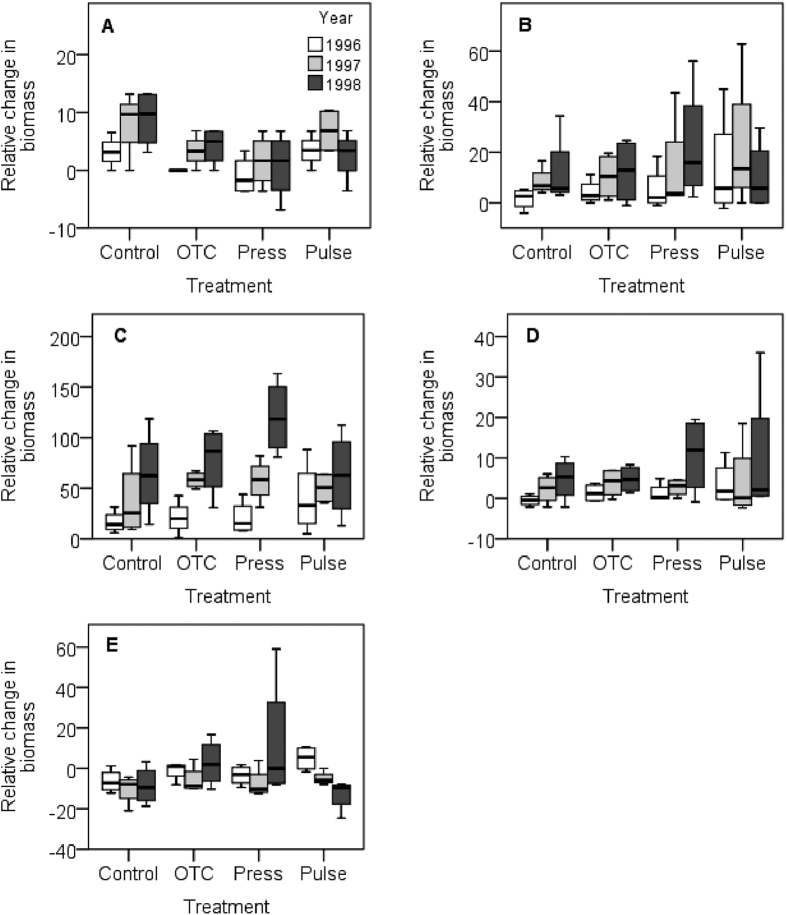
Boxplots of responses in relative change in the biomass of plant functional groups in the alpine meadow. (**A**) Cushions, (**B**) deciduous shrubs, (**C**) evergreens, (**D**) forbs, and (**E**) graminoids. Treatments: control (Control), constant warming enhancement using open-top chambers (OTC), a stepwise increase in the magnitude of warming (Press) and a single-summer high-impact warming event (Pulse). Boxplots show the 10^th^ to 90^th^ percentiles of the data; n = 4 plots per treatment.

Responses for the functional groups of vascular plants show that cushion plants and forbs responded significantly interms of cover, number of species, and biomass to the treatments (Table 2; Figs 3, 4, 5, Supplementary Dataset S2). All treatments tended to increase the cover and biomass of cushion plants over the years, with the pulse treatment causing a delayed positive response in 1997 to the 1996 season of experimental extreme warming (Figs 3 and 5). The treatments had contrasting effects on the number of species. The OTCs and pulse treatment had a positive effect, whereas the stepwise warming had a negative effect (Fig. 4). Forbs tended to increase in cover and biomass in all treatments over the years (Figs 3 and 5). The effect on the number of species of forbs also varied with treatments, with OTCs having a positive impact whereas stepwise warming a negative effect (Fig. 4). Significant responses to treatments were also observed among evergreens with respect biomass and among graminoids with respect to cover (Table 2, Figs 3 and 5). Significant responses to years were observed in cushions with respect to cover, in evergreens with respect to cover and biomass, in forbs with respect to biomass, and in graminoids with respect to cover, number of species, and biomass (Table 2, Figs 3, 4, 5). Interactions between treatment and years were observed in the number of species of forbs (Table 2, Fig. 4).

## Discussion

To our knowledge, this is the first climate change study to distinguish among the effects of constant, stepwise, and pulse warming on alpine/arctic vascular plant communities.

It is worth noting that the summers of 1996 and 1997 were abnormally warm, setting high-temperature records in consecutive years (at the time of the study). This unusual event may explain the positive development observed in the control plots as they were experiencing a “natural warming”. The unusually warm summers is a plausible explanation for the significant effects found with respect to year and the interaction effects among treatments and years.

At the canopy level, the experimental perturbations had a significantly negative effect on the number of species but not for Simpson’s D. At the bottom layer, we observed that the treatments had a significant effect on the number of species and Simpson’s diversity index. Whereas we found no first-year effect on species number from the pulse treatment, species number tended to decline in the two years following the pulse-warming event. As hypothesised, the stepwise increase in warming over the years resulted in a third-year effect, which caused a decline in Simpson’s D.

The impact on species richness was somewhat surprising, as many species in the high alpine and arctic regions are long lived. Long-lived species have also been suggested to be less sensitive to increased climate variability^34^. Thus, we had expected that the treatments would not cause a decline in species richness over the limited time of the study unless they had a severe negative impact on other plant traits, which did not seem to occur. However, in studies with a different experimental design, a decrease in species richness has been observed; for example, in bryophytes, lichens and forbs, a decline in richness was found to be caused by a loss of rarer species over a nine-year study^35^. Furthermore, sedges were found to decrease in response to warming in a seven-year study^23^. Thus, climate change may have a somewhat rapid impact on plant communities that are typically dominated by long-lived species, and the longevity of plants may not have the buffering effect that has been suggested.

At the community level, the only significant change that we found was in biomass, which increased significantly over the years and treatments. As hypothesised, this effect was most pronounced in the third year of the stepwise increase of warming. At the canopy level, the temperature perturbations had a significant effect on cover, biomass, and plant height. Higher plant production, in terms of increased biomass, plant height and cover, is a natural response to higher temperatures by plants that have temperature-limited growth, and similar responses have been reported in previous studies that have simulated climate change^36^. However, other experimental warming studies have presented somewhat contrasting results. For example, four years of warming using OTCs decreased biomass in a Canadian grassland^37^, and in a five-year experiment in a sub-arctic heath in Sweden, the authors found no effect on biomass from warming^38^. In addition, although not directly comparable, a study applying different levels of warming (high and low) over a five-year period on a sub-alpine heath and a high alpine fell field near Abisko, Sweden, found that higher levels of warming (by 4.9 °C) caused a significant increase in the biomass of vascular plants in the fell field^39^.

Canopy cover decreased slightly over the years in the pulse treatment, whereas the OTC and stepwise treatments had a positive effect on cover that increased over the years.

Simultaneously, the pulse treatment caused a dramatic increase in plant height and biomass in the first year, which then remained stable over the subsequent two years. Our results demonstrate that cover and biomass may show different trends and that stepwise treatments may induce accumulative responses to cover, biomass and plant height of the canopy cover. The accumulative trend was also supported, as the third-year increase was more pronounced in the stepwise warming treatment. Additionally, as hypothesised, stepwise warming caused a radical increase in both plant height and biomass in the third year.

Although not identical in experimental design and impact, our results may be compared to studies that have revealed contrasting effects from experimental heat waves, ranging from positive to neutral to negative impacts. For example, two different short-term pulse warming events in Greenland caused declines in average plant cover and an increase in total plant cover in response to 13 days or 8 days of warming, respectively^31^,^33^. In a study in Greenland they found no significant differences at the community level between plots experiencing two consecutive heat waves and the control plots, although dead plant material increased significantly in the heated plots^32^. These contrasting results from Greenland may partly be explained by the study design^33^. Similarly, little effect from heat waves *per se* was found in an experiment imposing heat waves and drought in Belgium; however, the combined effect of a heat wave and drought caused a decline in biomass^40^.

We are only aware of two studies that have measured the impact of extreme climatic events on the bottom layer of plant communities, both of which excluded vascular plants because they were focused on *Sphagnum* spp.^12^ and bryophytes and lichens^29^. Here, we included all bottom layer plants (vascular plants, bryophytes and lichens). In the present study, we found that the treatments had a significant effect on cover and biomass. As hypothesised, we found a first-year effect on the cover of the bottom layer in response to the pulse warming treatment; however, in contrast to our hypothesis, the stepwise increase in warming over three years caused an initial increase in biomass before returning to the starting level. Meanwhile, the OTCs caused no response. Furthermore, biomass tended to increase in response to all treatments among years.

Similar to cover, the pulse treatment caused the hypothesised first-year effect on biomass, which was an increase, and the biomass increase continued at a more limited scale over the subsequent two years. We also found a significant influence of year on cover and biomass in the bottom layer, which might have been an indirect effect of the unusually warm summers of 1996 and 1997. The warm summers may have contributed to the significant interaction effects between treatments and years with respect to cover in the bottom layer.

Our results are somewhat surprising considering the die-off of *Sphagnum* in the Italian Alps as a response to natural heat waves^12^. Our previous study that focussed on bryophytes and lichens in the same experiment revealed that experimental warming (collectively) had a significant impact at the community level and that pulse warming had a positive impact on the cover and biomass of acrocarpous bryophytes; however, at the species level, only a single pleurocarpous species, *T. nitens,* showed significant effects. Overall, bryophytes and lichens exhibited considerable resilience to short-term perturbations^29^. We believe that the differences in responses likely arose because the natural extreme events in the Italian Alps were accompanied by a drought, whereas the experimental study in alpine Sweden was unaffected by drought^29^. The importance of drought during extreme climate events has been shown in other studies as well^41^; hence, extreme warming events not accompanied by drought may not be detrimental to plant communities^29^.

Our results show that cushion plants responded positively in terms of cover, biomass, and number of species to the treatments. For this life form, the OTCs tended to increase the cover and biomass over the years, whereas the pulse treatment caused an initial positive response that continued the year following the pulse-warming event, after which the cover and biomass returned to the initial starting values. However, the most notable increase among years was found in the control plots that experienced unusually warm summers in 1996 and 1997. However, in 1998, their cover remained the same as during 1997, which suggests that they had taken advantage of the favourable growth conditions during the previous years. There are very few other experimental studies that have included cushion plants; however, the response of cushion plants can be compared with a study on *Silene acaulis*, a circumpolar cushion plant that was exposed to a factorial experiment with warming nutrient addition over a period of six years. In that study they showed that *S. acaulis* was able to respond rapidly in terms of vegetative growth and cover to the treatments; however, the initial positive response turned negative at the end of the study^42^. This finding demonstrates that although the species was able to respond rapidly when experiencing favourable conditions, it would likely become outcompeted in the long term if temperature and/or nutrient availability increased^42^. This response, in turn, could cause a cascading effect on ecosystem functioning, as cushion plants commonly function as foundation species, nurse plants, and facilitator species across trophic levels in severe environments^42^,^43^,^44^.

Forbs tended to respond positively in terms of cover and biomass to the treatments, and as hypothesised, the third year of the stepwise warming brought the largest increase in cover and biomass. We believe that the unusually warm summers of 1996 and 1997 brought about an increase in the control plots. The OTCs had a positive impact on the number of species, whereas the stepwise warming caused a negative effect. Graminoids decreased in cover in all treatments (including control plots) among years, with the greatest effect found in the pulse treatment, where they continued to decrease during the following two years after pulse event.

Significant responses to treatments were also shown by evergreens with respect to biomass, which increased in all treatments among years. As hypothesised, the response was most pronounced in the third year of the stepwise warming treatment. The unusually warm summers during the study period caused a natural “warming effect”, which was similar to the responses caused by OTCs in many cases. Our results support the previous findings that the natural warming in control plots caused significant effects in untreated plant communities^45^.

Above-ground increases in heath biomass have also been found during a nine-year study (1991–1999) in a nearby valley^46^. Additionally, a meta-analysis of control plots from 46 climate change experiments (ranging from 1980 to 2010) found that shrubs, graminoids and forbs all increased in height and that shrubs increased in abundance^47^.

However, such responses are not always the case, as a 20-year study involving a 2 °C ambient warming in northern Sweden found no change in vascular plant cover^48^. In Greenland, an experiment that imposed a heat wave over 13 days found that the forb *Polygonum viviparum* tolerated the heat wave better than the graminoid (sedge) *Carex bigelowii,* whereas the willow *Salix arctica* was the most sensitive^33^. Additionally, in a Swedish site in Abisko (near our field site), five years of high levels of warming (+4.9 °C) were found to cause contrasting effects on functional groups: evergreen shrubs increased their biomass significantly, whereas deciduous shrubs and herbs showed no significant response. At the same time, less rapid warming (+2.5 °C) caused no significant changes in biomass^39^.

## Conclusions

To summarise, this unique study shows the complex responses of the alpine plant community to different experimentally imposed climate-warming regimes. Plant cover, height and biomass frequently responded differently to a constant level of warming, a stepwise increase in warming and the extreme pulse-warming event. Notably, stepwise warming was found to have an accumulating effect on biomass. Furthermore, we show that the responses to the different warming regimes vary among functional groups and that short-term perturbations negatively affected species richness and diversity. As there are only a few experimental studies that have incorporated the impact of different warming regimes, there is a need for further studies to improve climate change models to be able to incorporate the impacts of larger variation in climate and more frequent climate extremes on plant communities, both of which are projected in the future.

## Materials and Methods

The fieldwork was conducted in northernmost Sweden at the Latnjajaure Field Station (LFS) in the valley of Latnjavagge, 68°21´N, 18°29´E, at an elevation of 1000 m. Since early spring 1992, a year-round automatic climate station has provided a continuous dataset. The valley is covered by snow for most of the year, and the climate is classified as sub-arctic^49^, with cool summers and relatively mild, snow-rich winters (annual minimum ranging from −27.3 to −21.7 °C) and a mean annual temperature of −2.0 to −2.7 °C (data from 1993–99). The annual precipitation ranges from 605 mm (1996) to 990 mm (1993); the mean for 1990–99 was 808 mm. July is the warmest month, with a mean temperature ranging from +5.4 °C (1992) to +9.9 °C (1997). The vegetation in the valley comprises a wide range of communities varying from dry to wet and poor and acidic to base-rich. Although the geographical location is subarctic-alpine, the vegetation of the region is representative of the Low Arctic, and the dominant species are *Cassiope tetragona*, *Dryas octopetala*, and *Carex bigelowii*^50^.

### Experimental design

The experiment for the present study was initiated in a rich meadow community approximately 300 m SE of LFS on a gentle NW-facing slope with an ample ground water supply. In July 1995, four blocks, each with four 1 × 1 m plots that were as similar as possible with regard to floristic composition and edaphic conditions, were marked and numbered. The different treatments were then randomly distributed in the four blocks (in 1995), and the actual treatments were initiated in June 1996, which enabled us to make a “before-impact” inventory at peak vegetation season in 1995. The treatments were (1) the control, (2) the standard OTC, (3) a stepwise increase of warming among years, and (4) a single season of pulse warming^29^. The standard OTC-experiments (2) followed Marion *et al.*^51^, using hexagonal polycarbonate chambers with a base diameter of 1 m^50^,^51^ that were fixed to the ground from early June 1996 to late August 1998. In the stepwise warming manipulation (3), an OTC was installed in the plot on 10 cm high pylons throughout the 1996 season, affixed to the ground throughout the 1997 season, and fitted with a polyethylene lid throughout the 1998 season, which increased the experimental warming each year^29^. In the pulse treatment (4), a closed-top chamber (CTC; a standard OTC provided with a polyethylene lid, as in (3)) was installed in the plot throughout the 1996 season and removed in late August the same year^29^. We used closed-top chambers for the pulse treatments because they have been used as experimental tools for studies on CO_2_ and H_2_O fluxes, evapotranspiration, photosynthesis and methane emissions in agricultural research^52^,^53^,^54^,^55^,^56^.

While the passive greenhouses did not allow for control of the temperature increase, they are robust (needed in the extreme environment). They also allowed us to impose different warming levels by manipulating the design slightly (by raising them from the ground for a smaller temperature increase, and closing the top for a greater temperature increase). The different treatments resulted in different warming levels. The temperature increase using the standard OTC remained at an average of 1.87 ± 0.25 °C (mean ± SE, n = 7 runs) above the ambient surface temperature in the adjacent control plots^29^. At the same time, the ventilated OTCs in the first treatment year in the stepwise warming treatment caused a more moderate increase of 1.00 ± 0.42 °C (n = 2), whereas the CTC treatment in the stepwise warming (year 3) and pulse treatments caused a greater increase of 3.54 ± 0.24 °C (n = 3). Thus, the treatments generated three different warming regimes for comparison^29^. The different experimental warming treatments can also be illustrated as three temperature units of ca. 1 °C each, where the OTCs (2 units) and stepwise warming (1, 2 and 3 units for the different years) had an equal cumulative sum (total of six units) after three years of treatment. However, the single-year pulse treatment (3 units) only had three units above the control for the same period^29^.

### Measurements

All sixteen plots were mapped in early August of each year (1995–98) in the same sequence such that each individual plot was mapped on roughly the same date every year. For mapping, a 1 × 1 m grid frame^57^ was used. In each of the 100 grid points, the specific identities of the topmost canopy (if present) and bottommost layer species (if present) were noted together with the height (1 cm accuracy) from the ground to the point of interception (canopy species only). In the square 1 × 1 m control plots, there were always 100 sampling points for the canopy and 96 points for the bottom layer, four points sacrificed for orientation screws with 5 mm head diameter, which enabled proper re-installation of the grid frame each year^57^. Due to their hexagonal shape, the OTCs reduced the number of points per plot to 87–94. Solifluction at the study site was very low and totalled less than 1 cm in horizontal distance over the four years of study.

The surface temperatures in some of the manipulated plots (always measured in comparison with the parallel control plots) were measured with Tinytag™ temperature loggers; the loggers recorded at 30 min intervals, and the series from which means were calculated comprised 1000–5600 timed readings each.

### Data analysis

The biomass of the various life forms was estimated using cover and plant height data according to the life form-specific algorithms established for the site^58^,^59^. The identification of life forms (functional types) followed Molau and Alatalo (1998). Cushion plants (e.g., *Saxifraga oppositifolia* and *Silene acaulis*) were treated as occupants of the bottom layer, and *Equisetum* spp. and *Selaginella selaginoides* were regarded as evergreen perennials. Diversity was measured as a combination of species diversity and relative frequency, which was calculated as Simpson’s Index of Diversity, D, according to D = 1 − Σf ^2^, where f is the relative frequency of a species (0 ≤ f ≤ 1). D values were corrected for sample size such that 0 ≤ D ≤ 1^50^.

### Statistical analyses

To investigate whether treatments and years significantly affected the different response variables (i.e., cover, number of species, species diversity, biomass, and height), we decided to use generalized linear mixed model analyses (GLMM) since it can include both fixed-effect factors and within-subject dependencies as random effects. We assumed that the block design (4 blocks) could cause causality in the analyses and we were not interested in analysing block effects *per se*. Block design was therefore included as a random effect in the GLMM models and thereby treated as random variation around a population mean (see e.g. Pinheiro and Bates, 2000). All data except cover were transformed prior to analyses (logarithmic and exponential transformations were used) to ensure there were no heterogeneity or over dispersion since that could influence the link-function and normal distribution conditions. The following four models were performed in GLMM: response variable ~ Treatment; response variable ~ Year; response variable ~ Treatment and Year; response variable ~ Treatment and Year with their interactions (Treatment * Year). Response variables were cover, number of species, species diversity, biomass, and height (height was only available for the canopy layer). Analyses were performed separately for the canopy and bottom layer since these represent different plant groups, the canopy layer consists of vascular plants whereas bryophytes, lichens and a few plants dominate the bottom layer. Furthermore, GLMM was performed for each (vascular) plant functional group, *i.e.* cushions, deciduous shrubs, evergreen shrubs, forbs and graminoids for the response variables biomass, cover and number of species. Akaike’s information criterion (AIC) was used for evaluating the quality of fit for the models. Model settings were normal distribution and identity link function, while the build options were at default. Only the model with the best quality of fit is presented. Analyses were performed in IBM © SPSS © Version 22.0.0.1.

## Additional Information

**How to cite this article**: Alatalo, J. M. *et al.* Impacts of different climate change regimes and extreme climatic events on an alpine meadow community. *Sci. Rep.* **6**, 21720; doi: 10.1038/srep21720 (2016).

## Supplementary Material

Supplementary dataset S1

Supplementary dataset S2

## Figures and Tables

**Table 1:** Results of GLMM for canopy and bottom layers for significant responses in cover (%), number of species, Simpson’s diversity index (Simpson’s D), biomass, and height (cm) to the experimental perturbations during three years at Latnjajaure Field Station, Northern Sweden.

Canopy Variable	Coefficient	P	Bottom Variable	Coefficient	P
**Biomass**			**Biomass**		
Treatment	n.a.	<0.0001	Treatment	n.a.	<0.0001
Year	n.a.	<0.0001	Year	n.a.	<0.0001
Treatment*Year	n.a.	0.002	1995	−0.098	<0.0001
Press	0.15	0.002	1996	−0.051	0.014
1995	−0.341	<0.0001	1997	−0.043	0.038
1996	−0.245	<0.0001	Control	0.125	<0.0001
1997	−0.145	0.002	OTC	0.047	0.023
			Press	0.114	<0.0001
**Cover**			**Cover**		
Treatment	n.a.	<0.0001	Treatment	n.a.	<0.0001
1996	9.15	0.047	Year	n.a.	0.036
OTC*1995	−15.75	0.018	1995	−16.3	<0.0001
OTC*1996	−14.78	0.024	Control*1995	12.28	0.023
Press*1995	−21.95	0.001	OTC*1995	16.93	0.002
Press*1996	−19.05	0.004	Press*1995	17.75	0.001
**Simpsons**		n.s.	**Simpsons**		
			Treatment	n.a.	0.029
			OTC	−0.185	0.02
**Number of species**			Press	−0.21	0.01
Treatment	n.a.	0.018			
**Height**			**Number of species**		
Year	n.a.	<0.0001	Treatment	n.a.	0.024
Treatment	n.a.	0.002	Control	0.085	0.029
Treatment*Year	n.a.	0.008	Press	0.11	0.005
1995	−1.113	<0.0001			
Press	1.165	<0.0001			
Press*1995	−0.87	0.021			
Press*1996	−1.51	<0.0001			
Press*1997	−1.232	0.001			

**Table 2:** Results of GLMM for plant functional groups and their responses in cover (%), number of species, and biomass to the experimental perturbations during three years at Latnjajaure Field Station, Northern Sweden.

Plant functionalgroup	Biomass			Cover			Number of species		
	Variable	Coeff	P	Variable	Coeff	P	Variable	Coeff	P
Cushions	Treatment	n.a.	<0.0001	Treatment	n.a.	<0.0001	Treatment	n.a.	<0.0001
Deciduous shrubs			n.s.			n.s.			n.s.
Evergreen shrubs	Treatment	n.a.	<0.0001	Treatment	n.a.	0.009			n.s.
	Year	n.a.	<0.0001	Year	n.a.	<0.0001			
	1995	−62.73	0.003	Press	12.65	0.013			
	Press	94.26	<0.0001						
	Press*1995	−57.54	0.049						
	Press*1996	−76.77	0.01						
									
Forbs	Treatment	n.a.	0.046	Treatment	n.a.	0.001	Treatment	n.a.	0.042
							Year	n.a.	0.007
							1995	−10.14	0.024
Graminoids	1996	17.84	0.013	Treatment	n.a.	<0.0001	Year	n.a.	0.022
	OTC	15.68	0.028	Year	n.a.	<0.0001	1995	1.5	0.046
	Press	20.65	0.004	1995	21.95	<0.0001			
	Press*1995	−25.6	0.012	1996	15.28	<0.0001			
	OTC*1996	−21.65	0.032	1997	6.9	0.041			
	Press*1996	−33.92	0.001	OTC	7.28	0.031			
	Press*1997	−28	0.006	Control*1995	−11.25	0.019			
				Press*1995	−11.28	0.019			
